# Correction: The purine nucleoside phosphorylase *pnp-1* regulates epithelial cell resistance to infection in *C. elegans*

**DOI:** 10.1371/journal.ppat.1010699

**Published:** 2022-07-07

**Authors:** Eillen Tecle, Crystal B. Chhan, Latisha Franklin, Ryan S. Underwood, Wendy Hanna-Rose, Emily R. Troemel

After publication of this article, the authors noted an error in the amino acid change predicted for the *pnp-1(jy90)* mutant allele. The mutant allele is incorrectly annotated as Leucine throughout the text. The wild-type codon is TCC, which codes for the amino acid Serine, and the correct *jy90* mutant allele is TTC, which codes for the amino acid Phenylalanine. The *PLOS Pathogens* editors have confirmed that this error does not affect the conclusions of the study.

Fig 1 is incorrect due to the error described above. The authors have provided a corrected version here.

**Fig 1:**
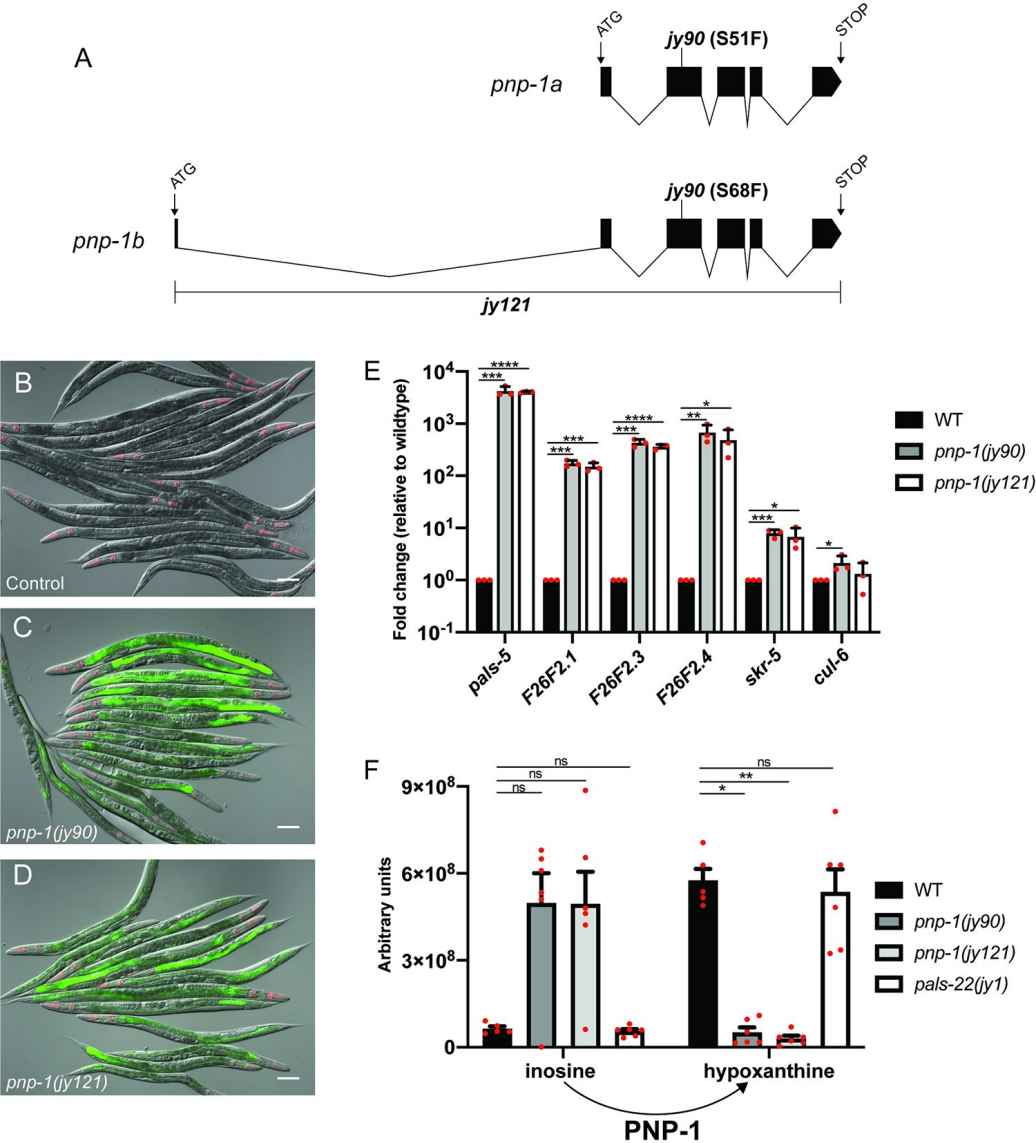
*pnp-1* mutants have increased expression of IPR genes. A) Gene structure of the two isoforms of *pnp-1* with exons indicated as black boxes. 5’ and 3’ untranslated regions are not shown. B-D) *pals-5p*::*gfp* IPR reporter expression in wild-type animals, *pnp-1(jy90)* and *pnp-1(jy121)* mutants. *myo-2p*::*mCherry* is a pharyngeal marker for the presence of the IPR reporter transgene. Scale bar is 100 μm. E) qRT-PCR of a subset of IPR genes in *pnp-1(jy90)* and *pnp-1(jy121)* mutants. Fold change in gene expression is shown relative to wild-type animals. Graph shows the mean fold change of three independent experiments. Error bars are standard deviation (SD). Mixed stage populations of animals were used. **** indicates p < 0.0001 by one-tailed t-test. F) Quantification of inosine and hypoxanthine levels in *pnp-1* and *pals-22* mutants from metabolomics analysis. Graph shows the mean levels of metabolites from six independent experiments for *pnp-1(jy121)*, *pnp-1(jy90)* and *pals-22(jy1)* mutants, and five independent experiments for wild-type animals. Error bars are standard error of the mean (SEM). ** indicates p < 0.01 by the Kruskal-Wallis test. E, F) Red dots indicate values from individual experiments. See materials and methods for more information.

In the *pnp-1* is a negative regulator of IPR gene expression subsection of the Results, there is an error in the fourth sentence of the first paragraph. The correct sentence is: From this analysis, we identified a missense mutation in the PNP gene *pnp-1*, which should result in substitution of a conserved serine (S51 or S68 in isoform a or b, respectively) to phenylalanine.

There is an error in the sixth sentence of the fifth paragraph of the Discussion. The correct sentence is: Support for the model that the catalytic activity of *pnp-1* is required for its effects on the IPR comes from the *pnp-1(jy90)* allele, which has a conserved serine mutated to phenylalanine.

In the Forward mutagenesis screening and cloning of *pnp-1(jy90)* subsection of the Methods, there is an error in the last sentence of the first paragraph. The correct sentence is: *pnp-1(jy90)* contains a G to A substitution that should convert serine 51 to phenylalanine in isoform A and serine 68 to phenylalanine in isoform B.
