# Supplementary figures and images for: The purine nucleoside phosphorylase pnp-1 regulates epithelial cell resistance to infection in C. elegans
Source: PLoS Pathog. 2021 Apr 20;17(4):e1009350. doi: 10.1371/journal.ppat.1009350 (PMC8087013; doi:10.1371/journal.ppat.1009350)

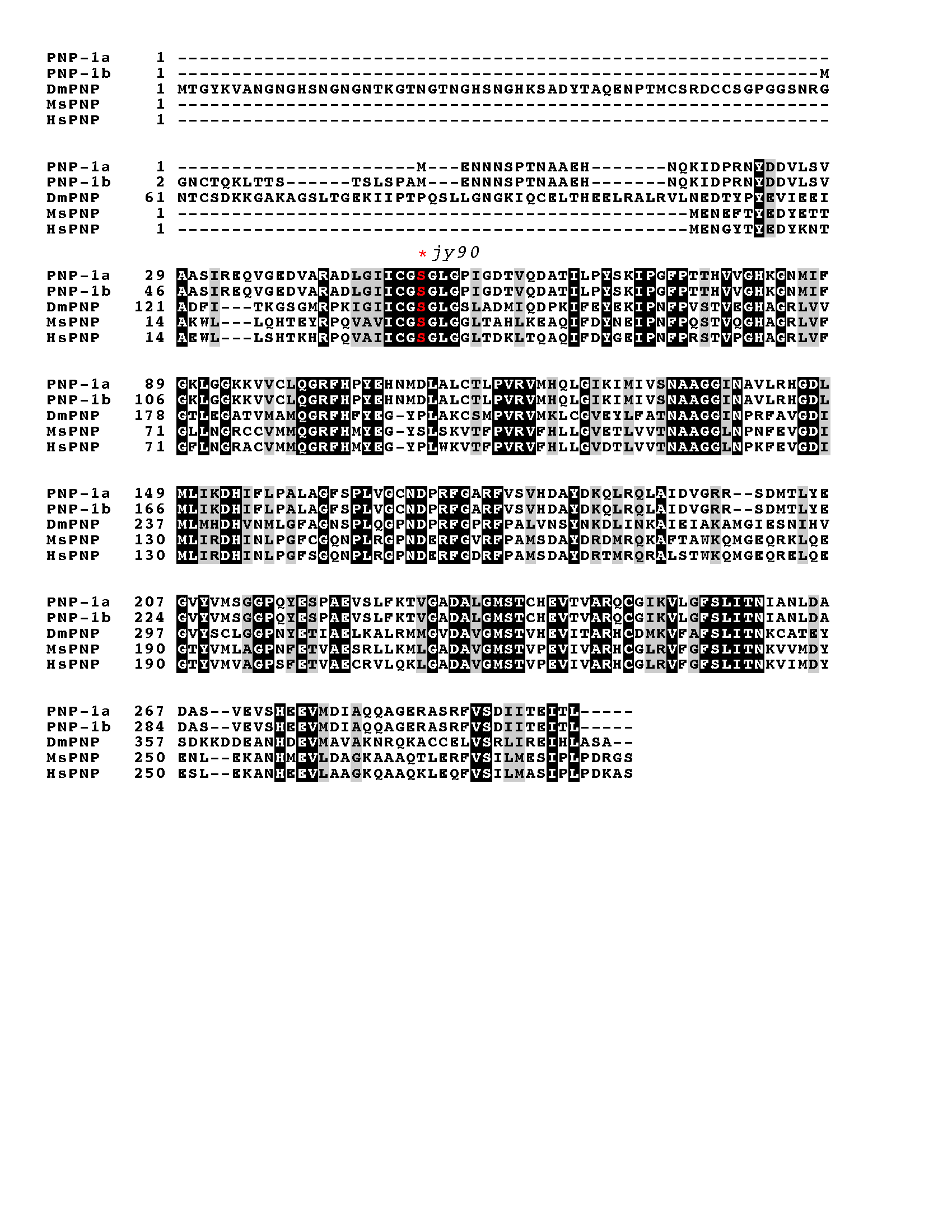

Supplement: S1 Fig — Alignment of PNP protein sequences from C. elegans (two isoforms, PNP-1a and PNP-1b), Drosophila melanogaster (DmPNP), Mus musculus (MsPNP) and Homo sapiens (HsPNP). Similar amino acids shaded gray, identical amino acids shaded black. Red indicates the serine converted to leucine in pnp-1 (jy90) mutants. Clustal Omega was used to perform the alignment (https://www.ebi.ac.uk/Tools/msa/clustalo/). BoxShade (https://embnet.vital-it.ch/software/BOX_form.html) was used to annotate sequence homology. (TIFF) [file ppat.1009350.s001.tiff]

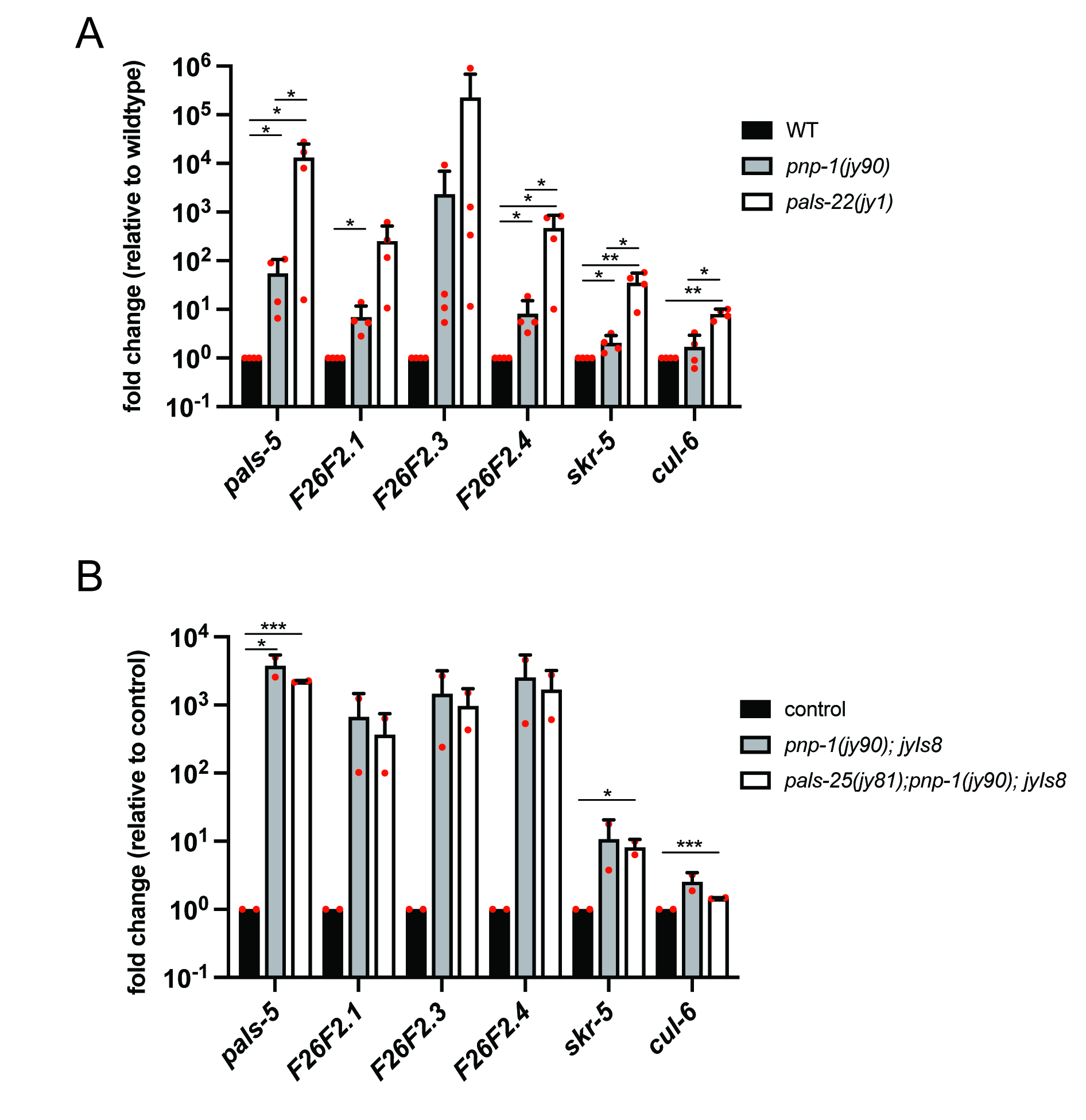

Supplement: S2 Fig — A) qRT-PCR of a subset of IPR genes in pals-22(jy1) and pnp-1(jy90) mutants. Synchronized animals grown for 44 hours at 20°C post L1 were used. Graph shows the mean fold change of four independent experiments. B) qRT-PCR of a subset of IPR genes in pnp-1(jy90) and pals-25(jy81); pnp-1(jy90) mutants. Mixed stage populations of animals were used. Graph shows the mean fold change of two independent experiments. A, B) Fold change in gene expression is shown relative to control animals. Error bars are standard deviation (SD). Red dots indicate values from individual experiments *** indicates p < 0.001 by a one-tailed t-test. (TIF) [file ppat.1009350.s002.tif]

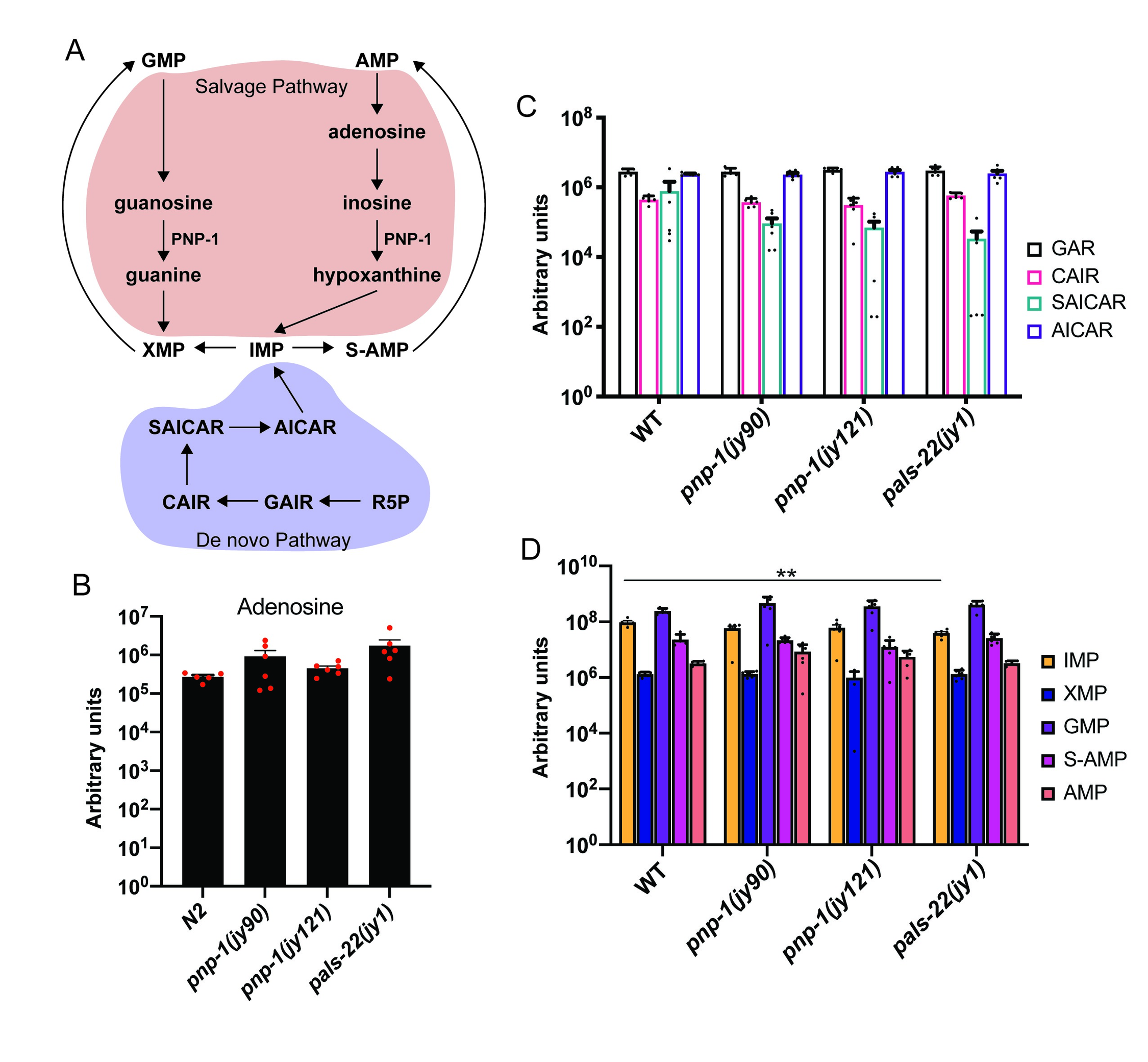

Supplement: S3 Fig — A) Schematic of purine synthesis pathways with select metabolites included. The salvage pathway is highlighted in red. The de novo pathway and metabolites are highlighted in blue. Metabolites that are not highlighted are common to both pathways. B-D) Quantification of adenosine, de novo specific metabolites and purine nucleotides, respectively (inosine and hypoxanthine are shown in Fig 1F). Graphs show the mean amount (in log10 scale) of the metabolites of six independent experiments for pnp-1(jy121), pnp-1(jy90) and pals-22(jy1) mutants and five independent experiments for wild-type animals. Dots (red or black) show individual values for each experiment. Error bars are SEM. Unless otherwise indicated, there is no significant difference in metabolite amounts in pnp-1 mutants or pals-22 mutants compared to control as determined by the Kruskal-Wallis test. ** indicates p < 0.01 by the Kruskal-Wallis test. Abbreviations used: R5P is ribose-5-phospate; GAR is glycineamide ribonucleotide; CAIR is 5’-phosphoribosyl-4-carboxy-5-aminoimidazole; SAICAR is succinylaminoimidazole carboxamide ribotide; AICAR is 5-Aminoimidazole-4-carboxamide ribonucleotide; IMP is inosine monophosphate; XMP is xanthine monophosphate; GMP is guanosine monophosphate; S-AMP is adenylosuccinate; AMP is adenosine monophosphate. (TIF) [file ppat.1009350.s003.tif]

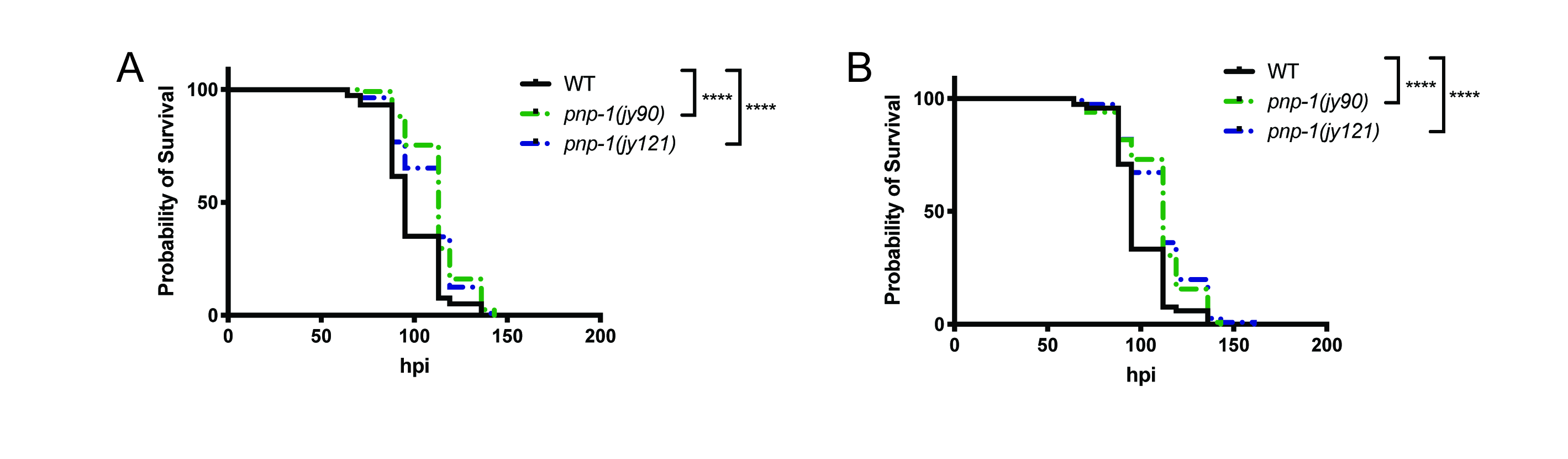

Supplement: S4 Fig — n = 120 per genotype for each replicate. **** indicates p < 0.0001 by the Log-rank (Mantel-Cox) test. (TIF) [file ppat.1009350.s004.tif]

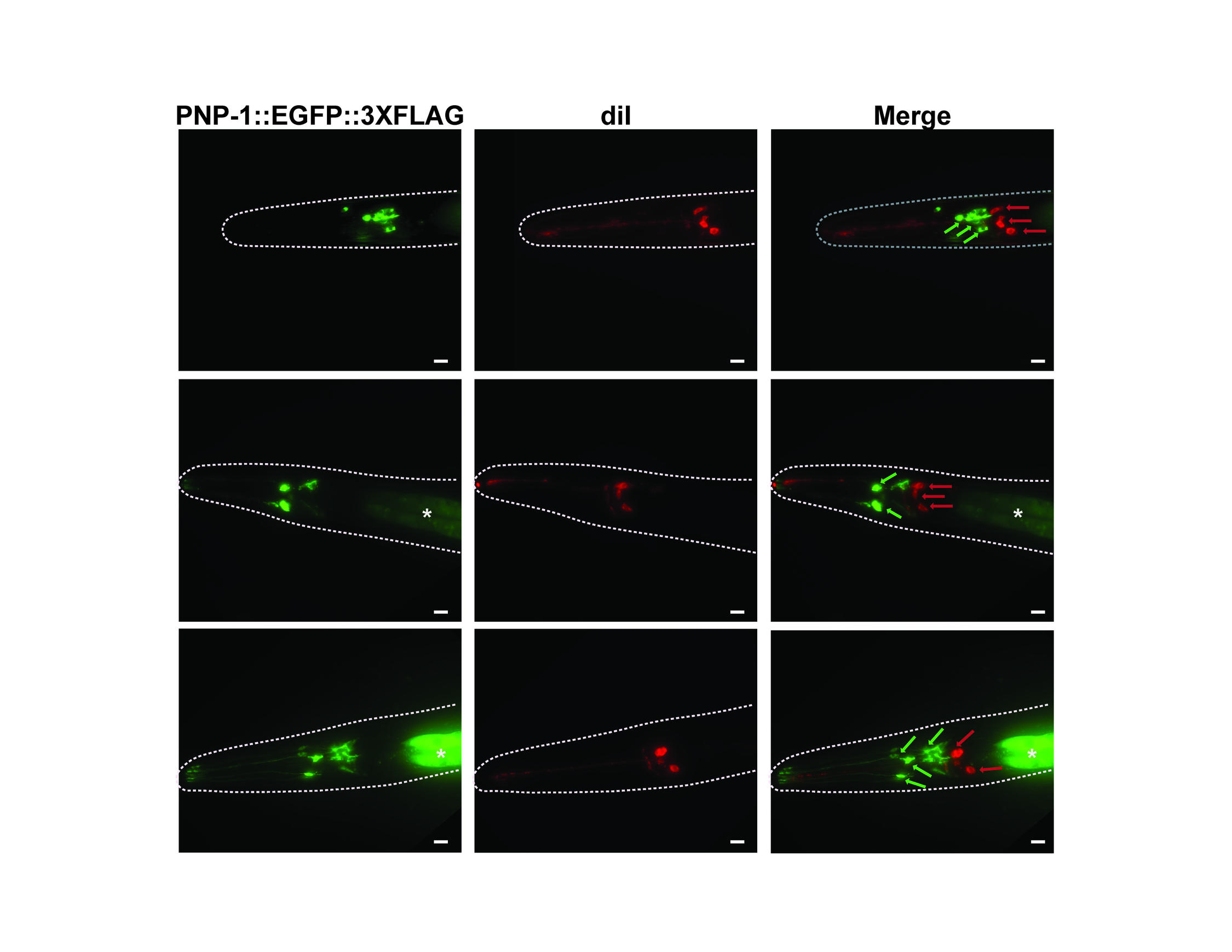

Supplement: S5 Fig — Transgenic pnp-1::egfp::3Xflag TransgeneOme animals, stained with the lipophilic fluorescent dye DiI, which labels a subset of amphid neurons in red. GFP-expressing cells (indicated by green arrows) are distinct from DiI-labled cells (indicated by red arrows), suggesting that pnp-1 is not expressed in the subset of amphid neurons that take up DiI. Each row is an individual animal and scale bar is 20 μm. The head of the animal is shown with anterior to the left. Worm bodies are outlined in white. Asterisks indicate GFP-expressing intestines. The exposure time for GFP in the bottom row is higher than that of the above two panels to better visualize the GFP-expressing processes extending anteriorly. (TIF) [file ppat.1009350.s005.tif]

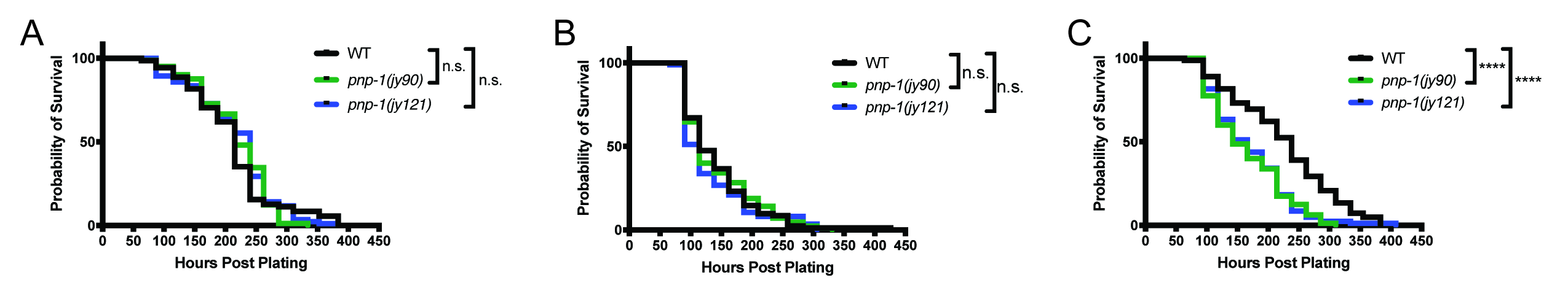

Supplement: S6 Fig — n = 90 per genotype for each replicate. **** indicates p < 0.0001 by the Log-rank (Mantel-Cox) test. (TIF) [file ppat.1009350.s006.tif]

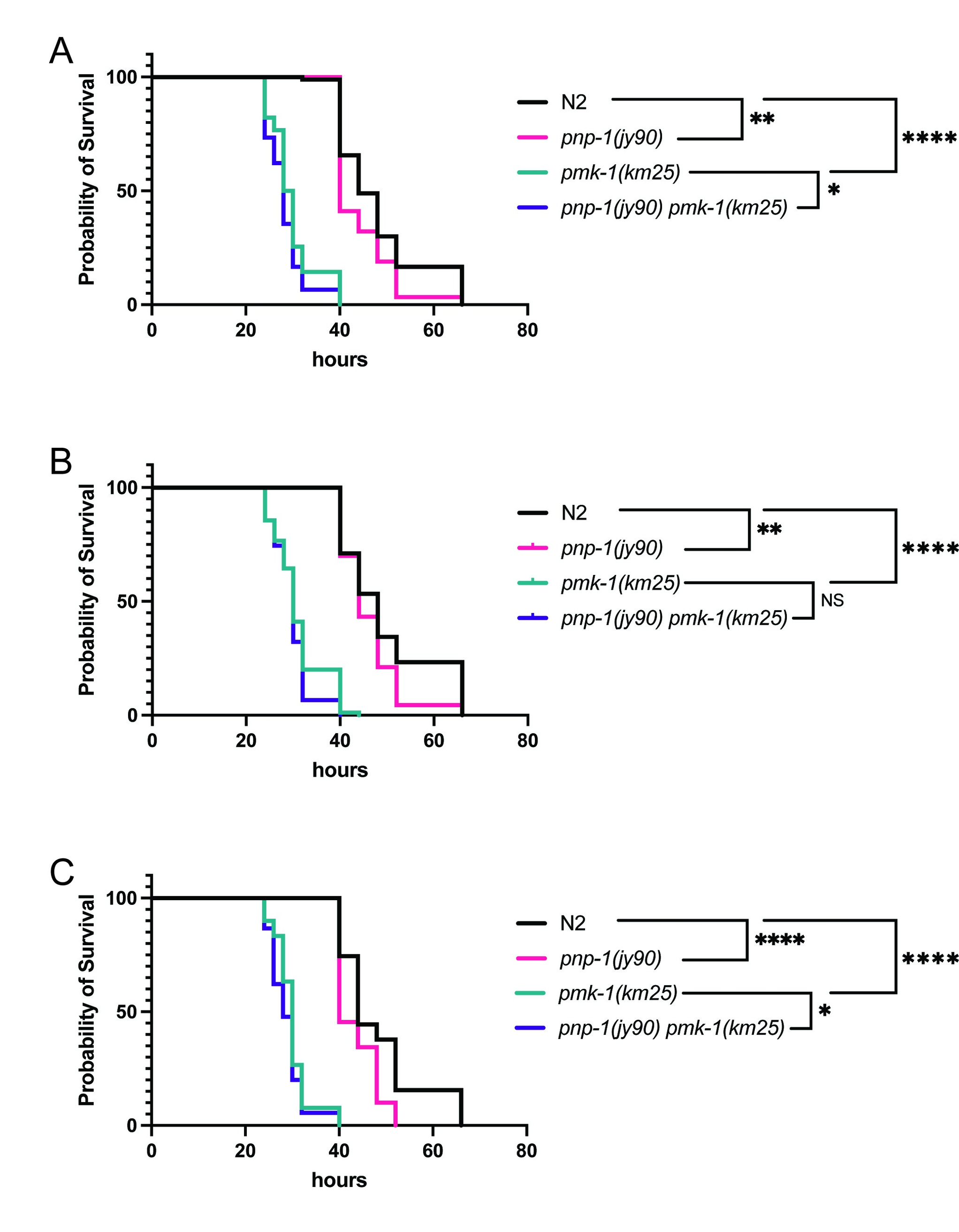

Supplement: S7 Fig — n = 90 per genotype for each replicate. **** indicates p < 0.0001 by the Log-rank (Mantel-Cox) test. (TIF) [file ppat.1009350.s007.tif]

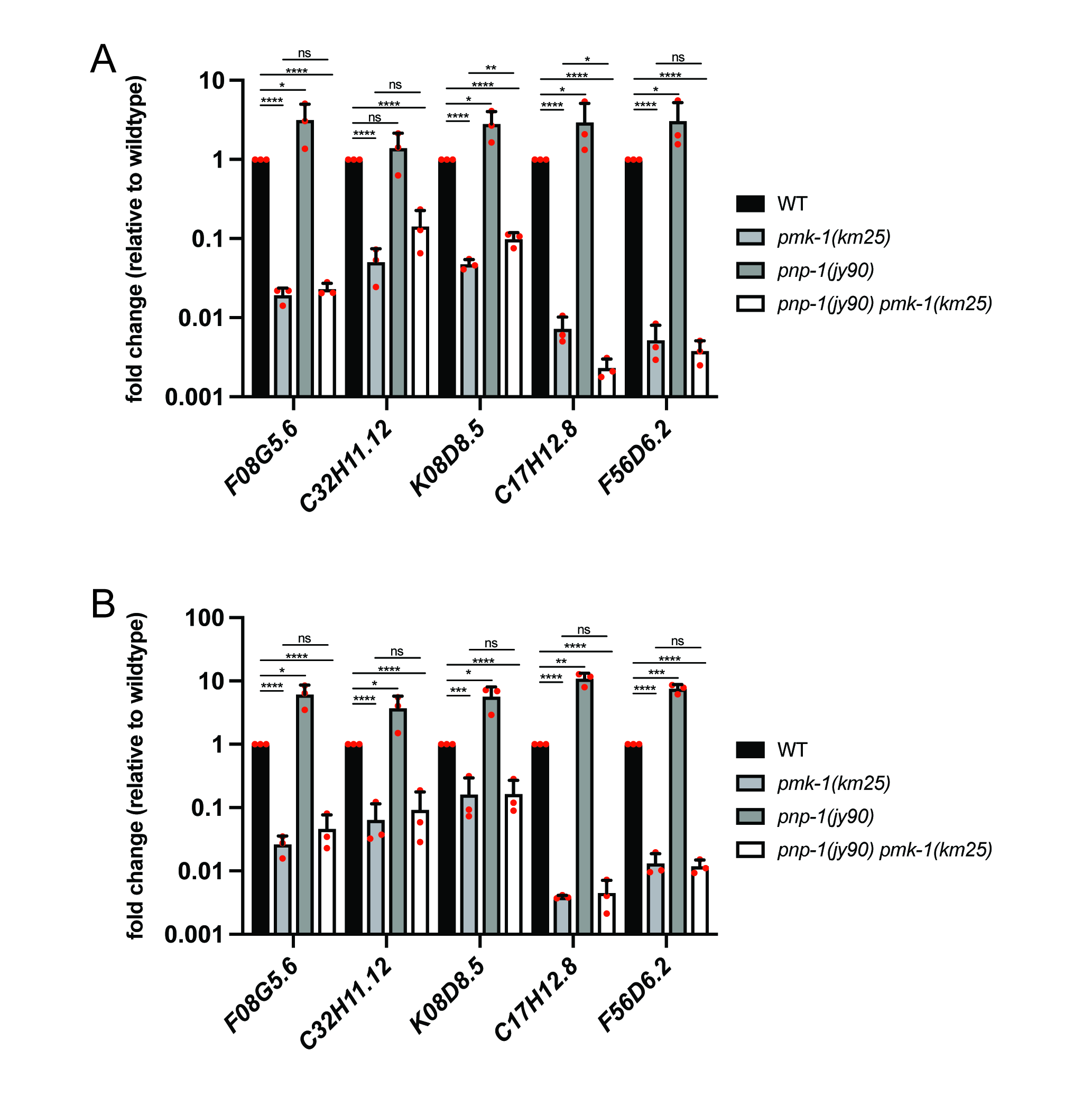

Supplement: S8 Fig — A) qRT-PCR of a subset of pmk-1 regulated genes in pnp-1(jy90), pmk-1(km25) and pnp-1(jy90) pmk-1(km25). Synchronized animals grown for 44 hours at 20°C post L1 were used. B) qRT-PCR of a subset of pmk-1 regulated genes in pnp-1(jy90), pmk-1(km25) and pnp-1(jy90) pmk-1(km25). Synchronized animals grown for 56 hours at 20°C post L1 were used. A, B) Fold change in gene expression is shown relative to control. Graphs show the combined results of three independent experiments. Red dots indicate values from individual experiments. **** indicates p < 0.0001 by a one-tailed t-test. (TIF) [file ppat.1009350.s008.tif]

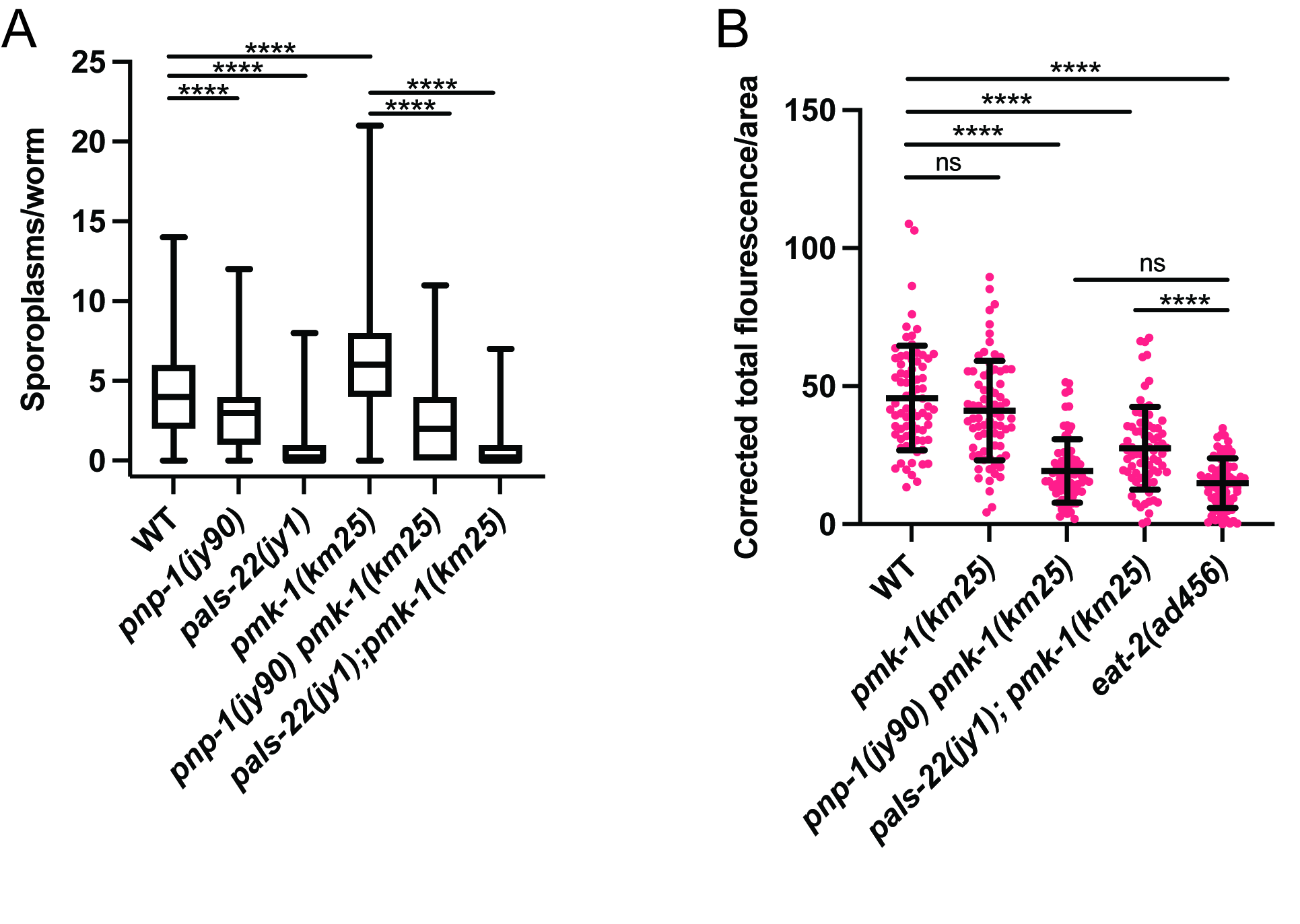

Supplement: S9 Fig — A) Quantification of N. parisii sporoplasm number in wild-type animals, pnp-1(jy90), pals-22(jy1), pmk-1(km25), pnp-1(jy90) pmk-1(km25) and pals-22(jy1); pmk-1(km25) mutants at 3 hpi. n = 400 animals per genotype. The box represents the 50% of the data closest to the median while the whiskers span the values outside the box. Graph shows combined results of four independent experiments. B) Quantification of fluorescent bead accumulation in wild-type animals, pmk-1(km25), pnp-1(jy90) pmk-1(km25), pals-22(jy1); pmk-1(km25), and eat-2(ad465) mutants. n = 150 animals per genotype. The corrected total fluorescence per worm was calculated and normalized to worm area. Each dot represents an individual animal. Graph shows combined results of three independent experiments. A, B) **** indicates p < 0.0001 by the Kruskal-Wallis test. (TIF) [file ppat.1009350.s009.tif]
