# Supplementary material for: The purine nucleoside phosphorylase pnp-1 regulates epithelial cell resistance to infection in C. elegans
Source: PLoS Pathog. 2021 Apr 20;17(4):e1009350. doi: 10.1371/journal.ppat.1009350 (PMC8087013; doi:10.1371/journal.ppat.1009350)
Supplement: Attachment — Submitted filename: 2021_03_27b_pnp1 response to reviewers.docx [file ppat.1009350.s020.docx]

**Response to Reviewers for Tecle et al**

We thank the reviewers for their time and effort in assessing our manuscript. We were grateful for the positive comments and the suggestions for improvement. See below for specific responses to each reviewer in blue, with their original comments in *italics*.

***********************

Reviewer #1:

We were pleased that this Reviewer commented, “*The findings presented in this manuscript are significant and of broad interest.*”

However, this Reviewer did note that the mechanistic basis for purine metabolism regulation of immunity is unclear. We agree with the reviewer, and this topic is an important area of research in the lab. Unfortunately we do not yet have positive results to report. However, we have included some negative results to the manuscript that address this topic, where we describe our efforts to add back purine metabolites and determine their effects on gene expression. See response to Major Issue #1 by Reviewer #2 for more detail.

**Major issues**

None

**Minor Issues**

*Figure 6: The authors suggest that the host may sense alterations in purine metabolism caused by pathogen infection as part of ‘surveillance immunity’. Here, perturbations in core cellular processes can lead to activation of pathogen defenses, like in pnp-1 mutants that exhibit alterations in purine metabolism and activation of the IPR and extracellular pathogen response genes. According to this model pnp-1 affects gene expression only indirectly. This does not become clear in the concluding model shown in Figure 6.*

We apologize for the confusion. In order to make this point more clearly, i.e. that PNP-1 regulation of gene expression is likely indirect, we have now added “Gene X” as an intermediary between PNP-1 and the IPR genes in Figure 6A.

*Also, shouldn’t the arrows from the pathogen infection point to purine metabolism rather than directly to the gene cassettes?*

We have now added an arrow from Orsay and Microsporidia infection to purine metabolism. We have also left the arrow directly from Orsay and Microsporidia to IPR genes; here we have strong support that the Orsay virus is detected through DRH-1 (the RIG-I-like receptor) sensing viral RNA (Sowa et al J Virology 2020), likely acting in parallel to purine metabolism.

*Spelling: Microsporisia infection > Microsporidia infection*

We have now made this correction.

*- Do pnpn-1 mutants exhibit increased survival upon infection with P. aeruginosa?*

We thank the reviewer for this question. We have now tested survival of *pnp-1* mutants and found that despite the decreased pathogen load in these mutants, they do not have increased survival upon infection, and in fact have slightly decreased survival. We hypothesize that this decrease is likely due to purine metabolism causing a decreased tolerance to bacterial infection in this mutant, perhaps similar to its decreased tolerance of heat shock. These new results are part of S7 Fig and described on p. 9, line 244-246.

*- All statistical tests used are parametric tests. Did the authors check if their data are really all normally distributed?*

We appreciate this question. We have now checked whether our data are normally distributed and have redone the statistics in the cases where we found that the data are not normally distributed. In almost all cases our conclusions still hold, but there is one difference. In particular, we found that the metabolomics data (n= 5 independent replicates) were not normally distributed and so redid this analysis. Here we found that hypoxanthine is still significantly lower in *pnp-1* mutants compared to wild-type animals, but inosine is no longer considered significantly different (although we note there is a large average increase in inosine levels in *pnp-1* mutants compared to wild-type animals – see Fig 1F). Thus, we have removed the word “significantly” from this sentence in the Results section. One exception where we haven’t modified our analysis is the qPCR data, as statistical tests tend to underreport significance in our experience, and the key results about gene expression changes have been validated with RNA-seq, showing statistics there.

Reviewer #2:

Reviewer #2 commented on the rigorous nature of our genetic analysis, and that *“The paper is also well composed: easy to read, the figures are clear, and the conclusions are well supported by the data. The information presented is to my knowledge new and provides an interesting new insight into the roles a highly conserved enzyme plays in the intra- and extracellular pathogen response.”*

***Major Issues***

*The paper describes the role of pnp-1 well, but some metabolite supplementation experiments might be able to better pinpoint what drives the gene expression and consequent organismal phenotypes. Specifically, the authors should test whether supplementation with hypoxanthine rescues some of the key defects of the pnp-1 mutants (or just one). This would reveal whether the defects arise due to lack of hypoxanthine (in which case they should be rescued) or due to build-up of inosine (in which case they might not be). One might hypothesize that the former would the the case as hypoxanthine availability might be limiting.*

*- Along those lines, what are the effects of hypoxanthine and inosine supplementation on gene expression. While RNA-seq is certainly outside of the scope of the manuscript, qRT-PCR of a few key genes (pals, etc) or even just a pals-5p::GFP reporter study in hypoxanthine-supplemented mutants and inosine-supplemented WT worms would reveal whether low hypoxanthine or high inosine drive the gene expression changes.*

We appreciate this suggestion from the reviewer. Indeed, we are keen to understand the mechanistic basis of *pnp-1* mutant phenotypes, and agree that lowered hypoxanthine or increased inosine are likely suspects. We have tried supplementation experiments to address these possibilities, alas with negative results so far. We now describe these supplementation experiments wherein we applied either inosine to *pals-5p::gfp* animals in a wild-type background to determine whether it would induce GFP expression, or hypoxanthine to *pnp-1;pals-5p::gfp* animals, to determine whether it would reduce GFP expression back to wild-type levels. In both cases there were no obvious changes, which are now reported in the Results section on p. 7 lines 156-163, with further details in Materials and Methods.

*- Further to this, the authors mention, but don’t discuss in detail, that no other metabolites in purine metabolism are changed. This is intriguing: how do the very substantial changes in inosine and hypoxanthine nevertheless not affect any of the other metabolites in this cycle? Does this reflect reduced rate in this metabolic pathway, i.e., steady state levels are the same but flux through the pathway is reduced (Fig S2 beautifully shows that there really are no apparent major detectable changes elsewhere)? I would be interested in the authors opinions on this.*

The reviewer raises an important point. It is somewhat curious that increased levels of inosine and decreased levels of hypoxanthine do not appear to lead to changes in other purine metabolites, at least among the species we measured. Here we speculate that because the measurements were performed in well-fed animals in a healthy state, that the de novo pathway is able to compensate for these changes. For future studies it would be interesting to determine whether *pnp-1* mutants have altered levels in other purine metabolites under stressful conditions, such as during infection or heat shock, perhaps because compensation with the de novo pathway is no longer possible when homeostasis is perturbed.

*- I’m also intrigued by the similarity between the pnp-1 mutants and the pals-22 mutant. How is this possible - do they regulate each other (unlikely as shown by metabolomics)? If not, what else can explain how a pnp-1 mutant results in significant similarity to the mutation in a pals gene? Again, I’d be interested in the authors opinion.*

We are also intrigued by the similarities between *pnp-1* and *pals-22* mutants. Here we speculate that there are many independent inputs to the IPR program, and that some aspect of purine metabolism controlled by *pnp-1* regulates IPR gene expression independently from the *pals-22* control of gene expression. Please see our response to Major Issue #2 from Reviewer #3, where we have performed additional analysis supporting this model of independent induction by *pnp-1* and *pals-25* (which is required for IPR activation downstream of *pals-22*). We have now added *pals-25* downstream of *pals-22* and independent of *pnp-1* in our Model in Figure 6, which hopefully makes this point about there being independent triggers of IPR induction – we are actively investigating how they converge on a common downstream transcription factor, which we plan to describe in a subsequent publication.

To provide more information on the similarity between *pnp-1* and *pals-22*, we have now included a new S4 Table that shows gene overlaps in these two mutants, as well as with IPR genes.

*- Fig 5 contains lots of information that could be mined some more. For example, the genes that are regulated in both pnp-1 mutants but not in the pals-22 mutant should be the ones that drive the extracellular pathogen response phenotype - can this be ascertained in some way? i.e. the authors also write that the pnp-1 genes are also significantly similar to genes regulated sta-1, skn-1, hsf-1, pmk-1 and sek-1, which in turn were different from IPR genes; this similarity should derive from that same gene set of 84 genes in Fig 5B - does this contain genes with e.g. predicted binding sites for any of these TFs and/or genes already known from other (screen) papers to confer resistance to infection with gram negative bacteria? etc.*

The reviewer makes an important point about the distinctions between *pals-22* and *pnp-1* mutants, both in terms of their phenotypes of gene expression and resistance to extracellular pathogens. One interpretation of our results is that the genes shown in previous studies to be induced by *P. aeruginosa* bacterial pathogens and regulated by known immune factors such as *pmk-1* mentioned above, may be responsible for the decreased *P. aeruginosa* pathogen load in *pnp-1* mutants as compared to *pals-22* mutants. We have tried to make this point more clearly on p. 9-10 lines 247-252, with new data in S8 Fig, showing that *pmk-1* is required for the increased expression of bacterial response genes in *pnp-1* mutants. This result is consistent with the finding that *pmk-1* is also required for the lowered pathogen load in *pnp-1* mutants.

In addition, we have now added a new table (S10) showing the overlap between genes induced in *pnp-1* mutants and those upregulated by *P. aeruginosa* infection and upregulated by wild-type *sek-1* and *pmk-1*. This is mentioned in the Results section on p. 12 on lines 327-334.

We also will note however, that upon further control experiments of *pnp-1 pmk-1* double mutants, we have found that they have lower accumulation of fluorescent beads compared to wild-type animals, as do the *pals-22; pmk-1* double mutants. We initially assumed that because each single mutant had similar bead accumulation to wild-type animals that the double mutants would have similar levels as well, but unfortunately that is not the case. Thus, we have moved the analysis of *N. parisii* pathogen load data in these double mutants from the main part of the paper (formerly Fig. 4F) into a new Supplementary figure (S9) right next to the new bead accumulation data. We state in the Results section on p. 10 line 260-269 what these results are and immediately follow with the caveat that the decreased pathogen load in the double mutants may simply be due to decreased exposure to *N. parisii*. However, because the double mutants in both cases have higher *P. aeruginosa* pathogen load than wild-type animals (and similar to the single mutants), we believe that the *P. aeruginosa* analyses are still valid because increased pathogen load here could not be explained by reduced pathogen exposure. We apologize for any confusion regarding these studies.

*- Methods: please, please deposit the RNA-seq results in NCBI GEO. This is a very interesting dataset and other investigators would greatly benefit from readily accessible raw data.*

The RNA-seq results have now been deposited in NCBI GEO. Accession number is listed on p.22 lines 644-645, and is GSE165786

**Minor Issues**

*In general it would be good if the authors would replace bar graphs with dot graphs or box and whisker graphs that better reflect the measurements. Fig 2 especially features graphs with large variation and it would be great to see individual data points there and elsewhere, as shown in Fig 2C and E.*

We appreciate the reviewer’s request for more data transparency. With the bar graphs shown in Figure 2A, we have now re-done the qRT-PCR with both *pnp-1* alleles. In both cases, we show with red dots the individual values for each experiment, as well as these values in bar graphs of other figures.

*- Fig 1E, the authors study several genes by qPCR. I noted the accession numbers F26F2.1, .3, and .4 - are these in an operon? If so, it’s natural that they display similar expression patterns.*

*Please clarify.*

While these genes are next to each other in the genome, they have not been reported to be in an operon together.

*- L167 "N. parsii”*

This has now been corrected.

*- Fig 3B may have an error with stats comparison, i.e. is the difference between pnp-1 mutant (2nd group) and non-rescued pnp-1 mutant really significant at **? Likely meant to be comparison between the latter two groups.*

We appreciate the reviewer’s careful attention to detail. In this case, there is a significant difference – the non-rescued *pnp-1* mutant is in an *unc-119* mutant background, and this mutation appears to have an effect on the pathogen load of this strain compared to the *pnp-1* mutant alone.

*Moreover, Fig 3B-C, the comparison between group 1 (WT) and group 3 (mutant rescued with WT) stats should also be shown.*

This comparison has now been added, as well as the comparison between *pnp-1* rescued and non-rescued animals in the *unc-119* mutant background.

*- L258 & Fig 5 the authors write "25 pals genes upregulated by N. parisii infection and Orsay virus, 22 are significantly up-regulated” but the figure shows only 13 genes.*

We have now clarified in the legend that only a subset of *pals* genes are shown in the figure. We apologize for the confusion.

Reviewer #3:

Reviewer #3 commented that *“the study is well-written and contains novel and convincing results that are likely to be generalisable to other systems.”* However, they noted some issues that we address below.

**Major Issues**

*The authors have shown that loss of pnp-1 leads to accumulation of inosine and activation of IPR in independent experiments. Can they show that direct supplementation of inosine is sufficient to trigger activation of IPR or make worms less susceptible to Orsay virus or N. parisii infection? The same also applies to supplementation of hypoxanthine, which is proposed to act as a repressor of pathogen response gene expression. Perhaps the authors have already tried these experiments so it may be good to discuss.*

We appreciate the reviewer’s comments here. We have tried the supplementation experiments but with so far negative results. Please see response to Reviewer #2 Major Issue #1 for more details.

*To strengthen their model that PALS-22 and PNP-1 act in parallel, can the authors show that the described IPR changes in pnp-1(-) mutants are not dependent on PALS-25?*

We thank the reviewer for this question. We have made a *pnp-1; pals-25* double mutant, and find that its IPR gene induction is similar to a *pnp-1* single mutant, supporting the model that *pnp-1* mutant phenotypes are not dependent on *pals-25*. These new results are part of S2A Fig and are described on p. 6 lines 130-133. Furthermore, PALS-25 is now included in the model figure in Fig. 6A.

**Minor Issues**

*Reviewer #3: The authors show that pals-22 mutants have higher resistance to viral/microsporidial infection than pnp-1 mutants. Is there any evidence that IPR induction in pnp-1 mutants is overall milder compared to that observed in pals-22 mutants based on qPCR (Fig. 1E) or RNAseq (Fig. 5)?*

Indeed, we have seen milder induction of IPR genes in *pnp-1* mutants compared to *pals-22* mutants. We now show this direct comparison in new data added to S2A Fig and described in the Results section on p. 6 lines 128-133. We also now allude to this finding in light of the stronger *N. parisii* resistance of *pals-22* mutants compared to *pnp-1* mutants on p.7 lines 178-181.

*It may be useful to show the overlap with IPR in the Venn diagrams in Figure 5A,B*

This overlap is now shown, and the genes are listed in new S4 Table.
